# A 28-Days Sub-Acute Toxicity Study in Swiss Albino Mice to Evaluate Toxicity Profile of Neurotol Plus (Mannitol and Glycerol Combination)

**Published:** 2009-12

**Authors:** Anupama Tamta, Manu Chaudhary, Rajesh Sehgal

**Affiliations:** *Intellectual Scientific Divison, R & D Centre, Venus Remedies limited, 51-52 Industrial Area, Panckula, Haryana, India*

**Keywords:** neurotol plus, mannitol, glycerol, repeated dose toxicity, intracranial pressure

## Abstract

Osmotic agents are still the most common treatment options available for controlling intracranial pressure (ICP). Combining Mannitol and Glycerol provides a better alternative and is currently available as best therapy used for increased ICP. The aim of this work was to study the effects of repeated dosing (28 days) of Mannitol 20% and Glycerol 10% combined formulation, Neurotol plus on safety profile. A twenty eight days sub-acute toxicity study was conducted at three different dose levels of 5ml/kg, 10 ml/kg and 20 ml/kg. Mice were randomly divided into four groups of six animals each. Physical parameters, biochemical parameters related to liver toxicity & nephrotoxicity and hematological parameters were studied as end point of evaluation. We also carried out histopathlogical studies to assess any organ specific toxicity. The present study demonstrated that there were either no or very minimal changes (at high dose) were observed on various physical, biochemical and hematological parameters between Neurotol plus and control group. In conclusion the data of present study suggest that the combination of Mannitol and Glycerol is not associated with any serious adverse effects and is safe therapeutic choice for ICP reduction.

## INTRODUCTION

Brain edema is one of the major causes of increased intracranial pressure (ICP), secondary deterioration, and death in patients after stroke attack (7, 12). Over the past few years, the use of previously recommended therapies such as barbiturates or hyperventilation has been increasingly questioned since they are known to reduce the cerebral perfusion pressure through negative effects on the systemic blood pressure or excessive cerebral vasoconstriction (12, 17). From that perspective, treatment with hypertonic fluids is still an attractive means of decreasing the intracranial pressure without having a negative effect on the cerebral perfusion pressure (8, 17). There are many reports supporting potential of substances like Glycerol and Mannitol in decreasing edema formation in brain (8, 10, 19).

Various clinical and experimental studies have demonstrated that single doses of Mannitol can substantially reduce increased ICP (2, 3, 14). However, the long-term beneficial effects of Mannitol are still controversial, and there are few reports of aggravated brain edema after repeated Mannitol treatment (8, 19). Glycerols are another attractive agent that has been found to exert beneficial effects in controlling ICP in edema and other pathological conditions. Apart from their hypertonic nature they also act as a free radical scavenger, antioxidant and activator of plasma prostaglandin resulting in vasodilation. Further, 10% Glycerol may improve ischemic brain energy metabolism as evident from available literature (13, 16, 23). These two agents i.e. Mannitol and Glycerol were combined as Neurotol plus to overcome lacunas associated with monotherapy using either agent.

Combination strategy enhances the diffusion of water from cerebrospinal fluid back into plasma by elevating the osmolality of the plasma (23). It rapidly enters the C.S.F. and brain compartments & favourably affects the recovery from stroke attack. The two mechanisms that may be responsible for this protective effect are redistribution of cerebral blood flow & regional cerebral blood volume and reduction in focal cerebral edema (16). Besides other advantages, Glycerol (10%) may become an alternative source of energy either by being directly metabolized by the brain or indirectly by enhancing lipogenesis or by both processes (3, 16) if glucose is lacking (10). Our unpublished data had reported this combination as better alternative to other drug of choice in patients with cerebral edema with or without hypertension or gastric ulcer.

Despite the facts that Mannitol and Glycerol are widely used to decrease elevated ICP and have many beneficial effects over other therapies, the safety has yet not been established on their long term uses. As the toxicity profiling is very much needed for the progress of any new formulation from preclinical to clinical stages. Considering that fact we carried out a sub-acute toxicity (28 day) study to assess safety profile of Neurotol plus (Mannitol 20% and Glycerol 10%) as combination regimen in Swiss albino mice.

## MATERIALS AND METHODS

### Animals

Healthy Swiss albino mice (male and female mice, 15–20 g weight) were divided into four groups (three treatment groups and one control group). Each group consists of 6 male and 6 female animals. Animals were provided with standard diet (pellets) supplied by Amrut feed India and water was given *ad libitum*. They were housed in polyurethane cages (three in each) at controlled room temperature of 25 ± 2°C and a relative humidity of 50.5% ± 5, and a constant light-dark schedule (12 hours light and 12 hour dark cycle).

### Reagents

Neurotol plus (Mannitol-Glycerol combination) was procured from Venus Remedies Limited.

### Experimental Design

Neurotol plus (Mannitol 20% + Glycerol 10%) was administered intravenously at three dose levels i.e. 5ml/kg, 10 ml/kg and 20 ml/kg body weight correspond to low dose, intermediate dose and high dose respectively for twenty eight days. Normal saline was administered to the animals of control group as sham treatment. Treatment was done once daily for 28 days. The study protocol for study was approved by Institutional Animal Ethics Committee (IAEC) of Institute for Toxicological Studies, Pune, India.

### Physical Parameters

Physical parameters (body weight, food and water intake), and local injury were studied throughout the treatment. Mortality if any, in all the groups, during the course of treatment was also recorded. Autopsy was done if animal died during course of treatment. At the end of treatment hematological, biochemical (liver function tests & renal function tests) and histological parameters were studied. The organs were quickly blotted, weighed on digital balance and processed for histological studies. The organ body weight ratio of each organ was calculated and tissues were processed for H & E staining.

### Hematological Parameters

Blood was collected by cardiac puncture. Blood samples were analyzed for routine hematological parameters. Blood cell counts were done with blood smears.

### Biochemical Parameters

Biochemical parameters were performed in plasma and serum. Serum gluatmic oxaloacetic transaminase (SGOT), serum gluatmic pyruvic transaminase activities (SGPT), serum levels of alkaline phosphatase (ALP), blood urea nitrogen (BUN), plasma protein and plasma sugar levels were estimated. All parameters were studied by Merck semi auto analyzer using Merck kits.

### Histological Examination

At the end of treatment animals were sacrificed and various organs like liver, kidney, lungs and gonads were collected for histological examinations. All the organs were immediately fixed in 10% buffered formalin and processed for histology with H&E staining.

### Statistical analysis

Results are shown as Mean ± SD. Significance of difference between groups was evaluated by using ANOVA. If ANOVA shows significant differences, post hoc analysis was performed with Dunnet test. P<0.05 was considered as statistically significant.

## RESULTS

The results of current study showed no adverse changes in physical parameters throughout the dosing period. There was no significant change in the mean body weight of the animals in Neurotol plus treated groups as compared to vehicle treated control group at the end of treatment (Table 1 and 2). The food and water intake of all the three groups were comparable to control group without having significant alteration in body weight and growth rate. No sign of local damage of tissue was observed at site of injection. No significant changes were observed in red blood cell (RBC), total leukocyte counts (TLC) and platelet counts in all the treated groups as compared to respective control groups (Table 3 and 4). The Haemoglobin level in Neurotol plus treated group of both sexes was significantly low in higher dose level as compared to control (Table 3 and 4). There were no significant changes that were seen in male and female mice groups total protein level in all the groups as compared to control group (Table 5 and 6). No significant increase was observed at even high dose level in serum glucose, SGPT and SGOT activities in all the treated groups as compared to respective control group. However BUN and Alkaline phosphatase level were found elevated at highest dose level of treated group (Table 5 and 6).

**Table 1:** Effect of sub acute doses of Neurotol plus on Physical parameter in male mice

Parameters	Organ	Control	Neurotol plus (5 ml/kg)	Neurotol plus (10 ml/kg)	Neurotol plus (20 ml/kg)

Body weight (g)		26.78 ± 1.53	24.88 ± 1.33	25.22 ± 2.15	25.82 ± 1.87
Food Intake/day (g)		3.1 ± 0.2	2.9 ± 0.5	3.0 ± 0.3	3.3 ± 0.5
Water Intake/day (ml)		4.1 ± 0.4	4.2 ± 0.2	3.9 ± 0.4	3.9 ± 0.7
Organ weight (g)	Liver (g)	2.54 ± 0.46	2.53 ± 0.54	2.61 ± 0.79	2.49 ± 0.49
	Kidney (g)	0.47 ± 0.03	0.48 ± 0.05	0.48 ± 0.03	0.49 ± 0.02
	Heart (g)	0.21 ± 0.02	0.20 ± 0.02	0.20 ± 0.01	0.22 ± 0.01
Organ body weight ratio (%)	Liver (g%)	9.61 ± 2.08	10.21 ± 2.36	10.30 ± 3.09	9.77 ± 2.45
	Kidney (g%)	1.78 ± 0.17	1.94 ± 0.19	1.90 ± 0.21	1.92 ± 0.12
	Heart (g%)	0.80 ± 0.10	0.82 ± 0.11	0.79 ± 0.08	0.86 ± 0.07

Values expressed as Mean ± SD, n=6.

**Table 2:** Effect of sub acute doses of Neurotol plus on Physical parameter in female mice

Parameters	Organ	Control	Neurotol plus (5 ml/kg)	Neurotol plus (10 ml/kg)	Neurotol plus (20 ml/kg)

Body weight (g)		25.95 ± 1.39	23.83 ± 2.62	23.88 ± 1.61	24.33 ± 2.33
Food Intake (g/day)		3.1 ± 0.4	2.9 ± 0.6	3.2 ± 0.4	3.3 ± 0.4
Water Intake (ml/day)		3.5 ± 0.6	3.6 ± 0.4	3.3 ± 0.7	3.8 ± 0.5
Organ weight (g)	Liver (g)	2.06 ± 0.67	2.82 ± 0.55	2.26 ± 0.57	2.55 ± 0.45
	Kidney (g)	0.48 ± 0.03	0.51 ± 0.02	0.49 ± 0.02	0.48 ± 0.05
	Heart (g)	0.23 ± 0.02	0.20 ± 0.02	0.23 ± 0.02	0.23 ± 0.03
Organ body weight ratio (%)	Liver (g%)	7.96 ± 2.65	12.06 ± 3.29	9.52 ± 2.60	10.88 ± 2.24
	Kidney (g%)	1.87 ± 0.13	2.14 ± 0.18	2.06 ± 0.11	1.97 ± 0.31
	Heart (g%)	0.87 ± 0.07	0.86 ± 0.13	0.97 ± 0.09	0.95 ± 0.14

Values expressed as Mean ± SD, n=6.

**Table 3:** Effect of sub acute dose of Neurotol plus on hemogram in male mice

Parameters		Control	Neurotol plus (5 ml/kg)	Neurotol plus (10 ml/kg)	Neurotol plus (20 ml/kg)

Haemoglobin (g%)		17.13 ± 1.68	15.67 ± 1.25	13.85 ± 1.10^a^	12.20 ± 0.75^a^
Total RBC (× 10^6^/cmm)		5.35 ± 0.79	6.02 ± 0.56	6.70 ± 0.45	6.80 ± 0.47
Platelets (× 10^5^/cmm)		8.47 ± 0.88	8.98 ± 0.53	8.85 ± 0.56	7.93 ± 0.78
Total WBC (× 10^3^/cmm)		5.37 ± 0.84	6.47 ± 0.61	6.15 ± 0.91	5.63 ± 0.48
Differential %	N	20.67 ± 2.80	19.33 ± 2.94	19.83 ± 2.32	18.50 ± 3.08
	L	76.50 ± 3.08	77.67 ± 2.88	77.67 ± 2.07	78.50 ± 3.99
	E	2.17 ± 0.75	2.27 ± 0.75	2.10 ± 0.75	2.15 ± 0.75
	M	0.67 ± 0.82	0.83 ± 0.75	0.33 ± 0.52	0.83 ± 0.75

Values expressed as Mean ± SD, n=6. RBC, Red Blood Cells; WBC, White Blood Cells; E, Eosiniophils; L, Leukocytes; M, Monocytes; N, Neutrophils;

a p<0.05, Vs Vehicle treated control.

**Table 4:** Effect of sub acute dose of Neurotol plus on hemogram in female mice

Parameters		Control	Neurotol plus (5 ml/kg)	Neurotol plus (10 ml/kg)	Neurotol plus (20 ml/kg)

Haemoglobin (g%)		16.73 ± 0.88	15.38 ± 0.95	13.77 ± 1.17^a^	11.82 ± 0.91^a^
Total RBC (×10^6^/cmm)		5.35 ± 0.90	6.17 ± 0.98	6.10 ± 0.82	5.73 ± 0.86
Platelets (×10^5^/cmm)		8.57 ± 0.73	8.50 ± 0.50	7.68 ± 0.83	8.27 ± 0.85
Total WBC (×10^3^/cmm)		5.90 ± 0.59	6.03 ± 0.56	6.47 ± 1.03	6.22 ± 1.02
Differential %	N	19.50 ± 2.43	20.00 ± 4.34	18.17 ± 2.71	19.00 ± 2.37
	L	77.33 ± 3.01	77.50 ± 4.81	79.00 ± 3.85	78.17 ± 2.64
	E	2.33 ± 0.82	2.00 ± 0.89	1.83 ± 0.98	2.33 ± 0.82
	M	0.83 ± 0.75	0.50 ± 0.55	1.00 ± 0.89	0.50 ± 0.55

Values expressed Mean ± SD, n=6. RBC, Red Blood Cells; WBC, White Blood Cells; E, Eosiniophils; L, Leukocytes; M, Monocytes; N, Neutrophils;

a p<0.05, Vs Vehicle treated control.

**Table 5:** Effect of sub acute dose of Neurotol plus on Biochemical parameters in blood of male mice

Parameters	Control	Neurotol plus (5 ml/kg)	Neurotol plus (10 ml/kg)	Neurotol plus (20 ml/kg)

Total Serum protein (g%)	6.10 ± 0.75	5.83 ± 1.37	6.00 ± 1.25	5.13 ± 0.77
BUN (mg %)	20.00 ± 2.10	22 ± 2.90	25.33 ± 2.73	28.83 ± 2.32^a^
SGPT (IU/L)	55 ± 6.07	56.50 ± 6.83	53.33 ± 5.50	54.50 ± 5.47
SGOT (IU/L)	90.33 ± 7.92	96.83 ± 7.44	104.83 ± 20.12	107.67 ± 16.29
SAP (IU/L)	247.17 ± 45.91	284.83 ± 34.16	344.67 ± 57.43^a^	387.50 ± 37.94^a^
Blood Sugar (mg%)	98.83 ± 4.88	101.50 ± 4.59	96.17 ± 7.88	96.00 ± 7.92

Values expressed as Mean ± SD, n=6. BUN, Blood urea nitrogen; SGOT, Serum gluatmic oxaloacetic transaminase; SGPT, Serum Gluatmic pyruvic transaminase activities; SAP, Serum Alkaline Phosphatase;

a p<0.05, Vs Vehicle treated control.

**Table 6:** Effect of sub acute dose of Neurotol plus on Biochemical parameters in in blood of female mice

Parameters	Control	Neurotol plus (5 ml/kg)	Neurotol plus (10 ml/kg)	Neurotol plus (20 ml/kg)

Total Serum protein (g%)	6.63 ± 0.64	5.83 ± 0.87	6.07 ± 1.30	5.42 ± 0.82
BUN (mg %)	20.50 ± 1.87	22.67 ± 1.75	25 ± 2.00	29.83 ± 3.66^a^
SGPT (IU/L)	54.00 ± 6.90	52 ± 5.76	56 ± 8.07	56.17 ± 5.49
SGOT (IU/L)	94.83 ± 8.8	96 ± 10.39	105.67 ± 15.73	102 ± 19.44
SAP (IU/L)	248.33 ± 42.86	301.17 ± 13.60	363.17 ± 53.30^a^	347.83 ± 49.50^a^
Blood Sugar (mg%)	98.83 ± 6.01	103.00 ± 3.69	101.50 ± 7.84	98.83 ± 4

Values expressed as Mean ± SD, n=6. BUN, Blood urea nitrogen; SGOT, Serum gluatmic oxaloacetic transaminase; SGPT, Serum Gluatmic pyruvic transaminase activities; SAP, Serum Alkaline Phosphatase;

a p<0.05, Vs Vehicle treated control.

Histological examination was done and there were no significant treatment related histopathological changes were observed in organs of all the treated groups. No damage was observed in the brain section of treated animals as compared to control animals. There was no mortality found till the completion of study.

## DISCUSSION

The aim of this study was to investigate the toxicity profile of multiple doses of Neurotol plus (20, 21). Mannitol (20%) is frequently used to reduce elevated intracranial pressure often associated with brain edema. Glycerol may reduce cerebral edema and increase regional cerebral blood flow. Furthermore, a beneficial metabolic effect on neural cell metabolism has been suggested by available reports (2, 3, 16). It was observed that, the combination of both these therapeutic principles is superior to the use of each substance alone (13). Limited data is available on safety profile of combination regimen. We therefore studied the toxic effects of Neurotol plus, a combination product.

The mechanisms by which hypertonic fluids act are still a matter of controversy. The traditional and still most widely accepted theory is that hypertonic fluids create an osmotic gradient between the intracerebral intravascular compartment and the cerebral parenchyma, resulting in dehydration and shrinkage of endothelial cells and brain tissue (21). For Mannitol, this effect has been repeatedly demonstrated in radiological studies in humans and in animal experiments (13, 21). Mannitol is a cell-impermeant, non metabolizable sugar administered intravenously as a hypertonic solution and has been used frequently for treating brain edema (2, 24). Clinical concentrations of Mannitol activate tyrosine and stress kinases and induce apoptosis in bovine aortic endothelial cells (19). Thus, the clinical use of Mannitol may exert direct deleterious effects on vascular endothelium by increasing oxidative stress. Glycerols act as a free radical scavenger, antioxidant and activator of plasma prostaglandin resulting in vasodilation (13). Glycerol also improve ischemic brain energy metabolism and act as source of energy for cells. In a recent report suggest that Glycerol infusion can decrease intracranial volume towards normal by dehydration of normal, but not damaged, brain tissue and protect against rebound phenomenon of Mannitol treatment (3, 6). Thus, combination regimen improved safety profile of Mannitol.

Our results indicate no physical changes that were observed during the study period of twenty eight days in all three Neurotol plus treatment groups as compared to control. Increase in body weights and growth of treated animals of either sex were of similar pattern as in control groups (Table 1 and 2). The food intake and water intake was showing similar pattern in all the groups including control and three treatment groups. Blood was evaluated for hematological toxicity, hemogram was estimated and results proved no significant changes on blood cell count in Neurotol plus treatment groups except at highest dose level. There was dose dependent lowering of haemoglobin levels in the treatment groups. The decrease in haemoglobin levels of mice treated with Neurotol plus may be due to the oxidative stress associated with mannitol therapy (18). Mannitol also caused increase in MAPK activity (22) as well as NF-kB activation (9) which may be another reason for deleterious effects seen at highest dose. The other blood parameters were unaffected with Neurotol plus treatment (Table 3 and 4). Kidney function related parameters estimated for renal impairment (21). Kidney function was evaluated by measuring various parameters. No significant differences were observed in glucose and proteins with respect to control (Table 5 and 6). There was significant change observed in BUN and SAP at highest dose level. The infusion of mannitol at higher doses has been earlier reported to deteriorate renal function, our results also showing similar pattern to available reports (4, 15). The increase in osmotic stress induced at highest doses of mannitol infusion may explain increase in SAP values.

The evaluation revealed that no group was more or less affected deleteriously by the treatment of Neurotol plus. It may be due to protective action of Glycerol (13). Neurotol plus had no effect serum alkaline phosphatase, SGOT and SGPT activities, which confirmed no liver function alteration by Neurotol plus treatment of either sex as compared to the respective control group (Table 5 and 6).

Available reports indicate that pre-treatment with 10% Glycerol or Mannitol has protective effects on delayed neuronal death in the gerbil hippocampal region (13). No morphological changes were observed in brain of Neurotol plus treated mice. Histopathological analysis had shown no signs of toxicity in any of organ in treatment groups as compared to Neurotol plus. Thus histopathological studies also confirmed the safety data of other physiological, biochemical and heamatological parameters after Neurotol plus treatment.

It appears to be indisputable that combination regimen of hypertonic solutions have not produced any deleterious effects on mice at two dose level. However minor changes were observed in few parameters at high dose level. This study provides clinically relevant data which can be utilised to decide the therapeutic safety of current dosage regimen. It can be concluded from the results of this study that combination therapy of Neurotol plus may provide a safe alternative for ICP and brain edema.
